# Improving Disease Gene Prioritization by Comparing the Semantic Similarity of Phenotypes in Mice with Those of Human Diseases

**DOI:** 10.1371/journal.pone.0038937

**Published:** 2012-06-14

**Authors:** Anika Oellrich, Robert Hoehndorf, Georgios V. Gkoutos, Dietrich Rebholz-Schuhmann

**Affiliations:** 1 European Bioinformatics Institute, Wellcome Trust Genome Campus, Hinxton, United Kingdom; 2 Department of Genetics, University of Cambridge, Cambridge, United Kingdom; Charité Universitätsmedizin Berlin, NeuroCure Clinical Research Center, Germany

## Abstract

Despite considerable progress in understanding the molecular origins of hereditary human diseases, the molecular basis of several thousand genetic diseases still remains unknown. High-throughput phenotype studies are underway to systematically assess the phenotype outcome of targeted mutations in model organisms. Thus, comparing the similarity between experimentally identified phenotypes and the phenotypes associated with human diseases can be used to suggest causal genes underlying a disease. In this manuscript, we present a method for disease gene prioritization based on comparing phenotypes of mouse models with those of human diseases. For this purpose, either human disease phenotypes are “translated” into a mouse-based representation (using the Mammalian Phenotype Ontology), or mouse phenotypes are “translated” into a human-based representation (using the Human Phenotype Ontology). We apply a measure of semantic similarity and rank experimentally identified phenotypes in mice with respect to their phenotypic similarity to human diseases. Our method is evaluated on manually curated and experimentally verified gene–disease associations for human and for mouse. We evaluate our approach using a Receiver Operating Characteristic (ROC) analysis and obtain an area under the ROC curve of up to . Furthermore, we are able to confirm previous results that the *Vax1* gene is involved in *Septo-Optic Dysplasia* and suggest *Gdf6* and *Marcks* as further potential candidates. Our method significantly outperforms previous phenotype-based approaches of prioritizing gene–disease associations. To enable the adaption of our method to the analysis of other phenotype data, our software and prioritization results are freely available under a BSD licence at http://code.google.com/p/phenomeblast/wiki/CAMP. Furthermore, our method has been integrated in PhenomeNET and the results can be explored using the PhenomeBrowser at http://phenomebrowser.net.

## Introduction

With the advent of whole-genome sequencing, researchers have focused on understanding the underlying molecular causes of hereditary human diseases to enable and improve their treatment. Genetic pleiotropy as well as the polygenic nature of some of the human genetic disorders create challenges in the quest of identifying causal genes for a disease. One important tool to understand human hereditary diseases are animal models. Animal models of a human disease do not only provide insights into the pathogenesis of the disease but also enable the evaluation of therapeutic strategies.

Over the past few years, large-scale mutagenesis projects have been proposed to systematically identify the phenotypes of organisms resulting from targeted modifications to the organisms’ genetic markup. Large-scale mutagenesis experiments provide a thorough examination of species’ phenomes and with that constitute the tantalizing possibility for revealing valuable information about the molecular mechanisms underlying human disease [1]. In particular, phenotype studies in mice have been demonstrated to provide insights into human disease mechanisms [2], [3].

One outcome of these experiments is the accumulation of large and rapidly increasing amounts of phenotype data. The biomedical community has responded to the challenge of providing methods for retrieving, analyzing and comparing the data by the introduction of phenotype ontologies. A large number of phenotype ontologies is now available for various species, including *Homo sapiens* [4], *Mus musculus* [5], *Caenorhabditis elegans* [6], *Drosphila melanogaster* [7] and *Saccharomyces cerevisiae* [8], to provide standardized and detailed phenotype descriptions within a species. The challenge we are currently facing is to integrate species-specific phenotype descriptions across the various species, thereby enabling the systematic analysis of phenotype information across species in order to understand the function of genes and their role in human disease [9].

Two approaches are currently in use to align species-specific phenotype ontologies. In the first approach, lexical mappings between the labels and synonyms of concepts in species-specific phenotype ontologies are used to identify related phenotypes in different species [10], [11]. The second approach towards integrating phenotypes across species relies on formal definitions of concepts in phenotype ontologies using the Phenotype Attribute and Trait Ontology (PATO) [12] and the Entity-Quality (EQ) syntax [9]. Using the second approach, a phenotype is decomposed into an affected *entity* and a *quality* that specifies how the entity is affected. The EQ representation allows for the phenotype definitions to be integrated across species following the application of automated reasoning over their combination with a cross-species anatomy ontology [9], [13]. This approach has been implemented in the PhenomeBLAST software and applied to the prioritization of candidate genes of disease [14].

Several methods have been developed to prioritize candidate genes for diseases using a variety of data, primarily relying on known gene–disease associations [15]. For example, the *GeneWanderer* approach [16] employs a distance measure on a protein-protein interaction network to identify gene–disease associations. Another system, *ENDEAVOUR* [17], utilizes a set of known genes to create profiles which are then used to find matching genes. *SUSPECTS* [18] prioritizes genes from a given chromosomal region, according to available gene and protein information, that might be implicated in a disease. Since most of the available tools rely on known gene–disease associations and follow a “guilt-by-association” approach [15], [19], [20], they cannot be applied to the prioritization of genes for diseases with yet unidentified molecular origins. However, information about phenotypes may be used to prioritize or predict candidate genes for diseases as well as functional relations between genes and proteins even in the absence of knowledge about the molecular basis of a disease [21], and approaches based on the integration of phenotypes across species were successfully applied to suggest gene candidates for diseases [13], [14].

Here, we present a method to prioritize candidate genes in mice based on comparing experimentally derived phenotype data with phenotype descriptions of human diseases. We apply our method to the collection of phenotypes available from the Mouse Genome Informatics (MGI) [22] database and compare those to the disease phenotypes available from the Online Mendelian Inheritance in Man (OMIM) database [23]. We evaluate our method using a Receiver Operating Characteristic (ROC) curve and achieve an Area Under Curve (AUC) of up to 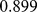. Our results demonstrate that our method significantly outperforms previous phenotype-based approaches of prioritizing gene–disease associations incorporating mouse model data (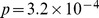 and 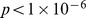, one-tailed Student’s *t*-test). Furthermore, we are able to provide evidence that *Vax1* (MGI:1277163) is involved in *Septo-Optic Dysplasia* (OMIM:#182230) and suggest *Gdf6* (MGI:95689) and *Marcks* (MGI:96907) as novel candidates. Our software as well as the data we produced are freely available from http://code.google.com/p/phenomeblast/wiki/CAMP.

## Materials and Methods

### Ontology Resources

In our approach, we incorporate the Mammalian Phenotype Ontology (MP) [5] as well as the Human Phenotype Ontology (HPO) [4] to analyze and integrate phenotypes. We obtained an MP version from the OBO Foundry ontology portal [24], last modified 21 June 2011. The version we downloaded comprised of 8,658 concepts. Furthermore, we obtained the HPO from http://www.human-phenotype-ontology.org. The version we used was last modified on 26 June 2011 and contained 10,282 concepts.

### Databases Containing Gene–disease Associations and Phenotype Information

We used two established resources containing gene–disease associations: the Mouse Genome Informatics (MGI) database [22] and the Online Mendelian Inheritance in Man (OMIM) [23] database. Both databases are populated by curators who manually extract the relevant information from the literature and report the information in a consistent framework.

The MGI database integrates genetic, genomic and phenotypic information about the laboratory mouse [22]. We used three report files from the MGI database (all accessed on 9 March 2011):

MGI_GenoDisease.rpt, MGI_GenePheno.rpt and HMD_Human5.rpt. The first report contains associations between diseases and the genotypes exhibiting the disease phenotype. Moreover, the report contains all genes that are targeted in the mutant mouse model that is associated with the disease. The second report contains the information about genotypes and their observed phenotypes. The phenotypes are represented using the MP. The third report file covers the information about human–mouse orthologous genes.

The OMIM database collects information about human heritable diseases, including genotype and phenotype information and known gene–disease associations. The version from 29 November 2010 contained 20,267 entries in total, out of which 13,606 described genes and over 7,000 described diseases [23]. To incorporate the OMIM information into our study, we obtained the MorbidMap file on 1 March 2011, available via the database’s download services. MorbidMap contains the information about known associations of human diseases and genes. The version we used, contained 2,717 diseases that were linked to 2,266 genes, with 3,463 distinct gene–disease associations (on average 1.27 genes per disease). The phenotypes associated with diseases described in OMIM are available as HPO annotations from the HPO web site (http://www.human-phenotype-ontology.org). The downloaded file comprised annotations for 5,027 OMIM entries.

### Ontology Mappings

An ontology is a specification of a conceptualization of a domain [25]. Ontologies consist of a set of concepts and relations as well as axioms that characterize the intended meaning of the concepts and relations. A *mapping* between two ontologies is a set of axioms that formally inter-relate the concepts and relations belonging to both ontologies.

We focus on mappings where the axioms relating concepts from two ontologies take the form of sub- and equivalent-classes axioms between atomic concepts. In particular, given the two concepts 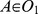 and 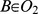, a mapping involving both *A* and *B* will be of the form:

- A SubClassOf: B, or
- B SubClassOf: A, or
- A EquivalentTo: B.

For a concept 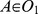, we will say that *A* maps to the concept 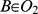, if *A* is either equivalent to *B* or a subclass of *B*.

### Mappings Through Lexical Matching

One approach to generate mappings between ontologies is to perform lexical matching on the labels (including synonyms) of concepts in ontologies [26]. We used the Lexical OWL Ontology Matcher (LOOM) [10] to generate a set of lexical mappings between concepts. LOOM generates a match between two concepts if either the concepts’ labels or synonyms can be matched lexically with at most a single mismatching character. Using LOOM on the HPO and MP ontologies, we extracted 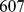 pairs of corresponding HPO and MP concepts.

Due to the use of lexical matching on concept labels, we assume that the 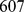 pairs represent *equivalent classes* axioms. For example, LOOM generates a match between the HPO concept *Melena* (HPO:0002249) and the MP concept *melena* ( MP:0003292), and we assume that this match represents the OWL axiom:

HPO:0002249 EquivalentTo: MP:0003292.

**Figure 1:**
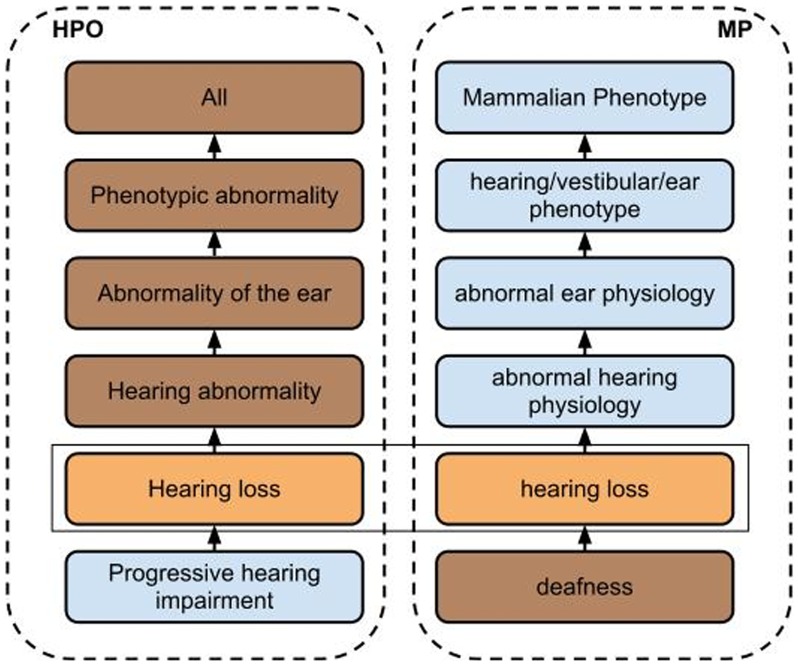
Illustration of an example mapping based on lexical matching. Concepts on the left side belong to HPO and all the concepts on the right side belong to MP. Applying LOOM to both ontologies extracted a lexical match between the HPO concept *Hearing loss* and the MP concept *hearing loss*. Based on this lexical match, the HPO concept *Hearing loss* is declared to be equivalent to the MP concept *hearing loss*, and the mapping for HPO’s concept *Hearing loss* will include the MP concepts *hearing loss*, *abnormal hearing physiology*, *abnormal ear physiology*, *hearing/vestibular/ear phenotype* and *Mammalian Phenotype*. The HPO concept *Progressive hearing impairment* will be mapped to the same MP concepts as *Hearing loss*. Conversely, both the MP concepts *hearing loss* and *deafness* are mapped to the HPO concepts *Hearing loss*, *Hearing abnormality*, *Abnormality of the ear*, *Phenotypic abnormality* and *All*.

For each match generated with LOOM, we added the resulting equivalent classes axiom to a knowledge base consisting of both MP and HPO. We then used an automated reasoner to classify the resulting ontology and with that generate a mapping from HPO to MP and a mapping from MP to HPO. To extract the mapping from HPO to MP, we iterated through all concepts in the HPO and performed a query for all classes that are equivalent to or are a super-class of the HPO concept and belong to MP. For example, the HPO concept *Progressive childhood hearing loss* is mapped to the MP concepts *hearing loss*, *abnormal hearing physiology*, *abnormal ear physiology*, *hearing/vestibular/ear phenotype* and *Mammalian Phenotype* based on the lexical match between the HPO concept *Hearing loss* (a parent-concept of *Progressive childhood hearing loss*) and the MP concept *hearing loss*. The example is illustrated in Figure 1. The mappings from MP to HPO were generated equivalently.

#### Mapping through automated reasoning

Mappings based on formal definitions were obtained using automated reasoning over anatomy and phenotype ontologies. For this purpose, we used the mappings generated by the PhenomeBLAST software for the PhenomeNET cross-species phenotype network [14] available at http://phenomeblast.googlecode.com. PhenomeBLAST integrates the formal definitions that were created for concepts from HPO and MP [9], [27], Gene Ontology (GO), UBERON [13], Mouse Anatomy Ontology [28], Foundational Model of Anatomy (FMA) [29], Mouse Pathology (MPATH) ontology [30] and Chemical Entities of Biological Interest (ChEBI) ontology [31] into a single ontology using a method for combining anatomy and phenotype ontologies [32]. The ontologies are then converted into OWL EL to enable efficient automated reasoning [33], and the CB reasoner is used to classify the resulting ontology [34]. To generate the mappings from MP to HPO, PhenomeBLAST identifies all equivalent and super-classes of an MP concept in HPO, and *vice versa* for the mappings from HPO to MP.

#### Combination of mappings

**Figure 2:**
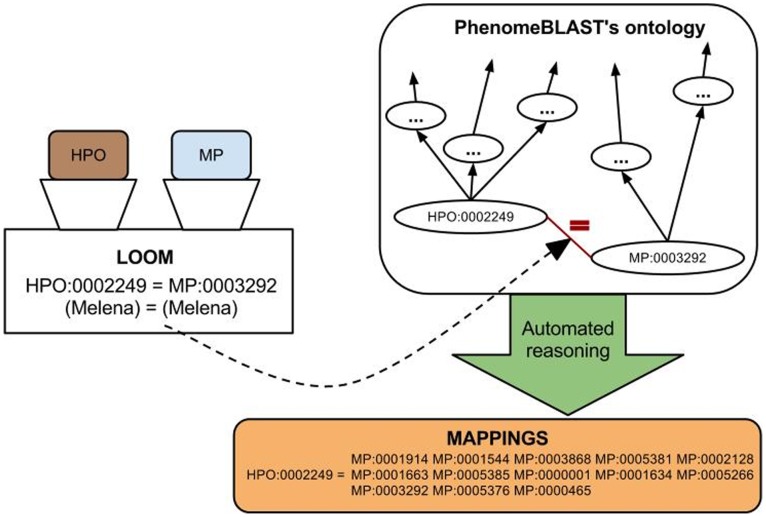
Integration of lexical and ontological mapping. The Lexical OWL Ontology Matcher is applied to the Human and Mammalian Phenotype Ontology to extract lexically matching concepts (based on labels and synonyms). All pairs of lexically matching concepts are inserted as equivalence class axioms into PhenomeBLAST’s ontology. A mapping is generated by reasoning over PhenomeBLAST’s adapted ontology and extracting all equivalence and super classes for each concept.

Since both the approaches to generate mappings between MP and HPO differ substantially, we combined both approaches and generated a novel mapping based on the formal definitions for concepts in phenotype ontologies and the lexical matches between the concepts’ labels and synonyms. We modified the PhenomeBLAST software to add the additional equivalent classes axioms derived from the lexical matching to PhenomeBLAST’s underlying ontology and used the modified PhenomeBLAST ontology to re-generate mappings between HPO and MP using automated reasoning. The process of combining both mapping approaches with each other is illustrated in Figure 2.

As a result, we obtain three different mappings from HPO to MP, which we call *lexical*, *ontological* and *merged*, and three additional mappings (lexical, ontological, merged) from MP to HPO, resulting in six mappings:

1. two mappings based on lexical matching (from concepts in the HPO onto concepts in MP and another mapping from concepts in MP onto concepts in the HPO),
2. two mappings based on automated reasoning over the concept definitions in phenotype ontologies (from HPO to MP and from MP to HPO), and
3. two mappings that combine automated reasoning over concept definitions in phenotype ontologies with lexical matching.

The mappings associate either a concept from MP with a set of HPO concepts or one HPO concept with a set of MP concepts. Using the ontological mappings generated through PhenomeBLAST, concepts from HPO are, on average, associated with 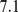 concepts from MP and MP concepts with 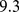 HPO concepts. Through lexical matching (using LOOM), HPO concepts are associated, on average, with 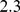 MP concepts and MP concepts with 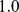 concepts from HPO. When combining the mappings, the average number of mapped concepts increases to 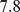 concepts from MP that are associated with an HPO concept and’ 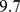 HPO concepts that are associated with an MP concept. Mapping through lexical matching produces, on average, significantly less concepts; for 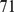
% of the concepts in HPO, we were unable to identify any corresponding MP concepts through the lexical matching approach, and similarly for 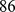
% of the MP concepts, no corresponding HPO concept could be identified.

To compare the obtained mappings directly with each other, we determined the overlap of the mappings obtained by either method given that a mapping for one particular concept was obtained using either method. Due to non-symmetrical mappings, we independently assessed both the “translation” directions: HPO to MP and MP to HPO. While comparing the results, we could identify four different categories the results fall into: exact overlap of the mappings, the lexical mappings are a subset of the ontological mappings, the ontological mappings are a subset of the lexical mappings, and the lexical and the ontological mapping overlap for a number of mapped concepts but each possesses also concepts not contained in the other. The coverage of the obtained overlap categories is shown in table 1.

**Table 1:** Illustrates the amount of mappings falling into each of the overlap categories when both methods are compared.

	HPO to MP	MP to HPO
# exact	110	93
# lexical 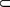 ontological	1367	502
# ontological 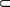 lexical	226	88
# overlap	1316	568
# concepts	3019	1251

Due to the mappings between HPO and MP not being symmetrical, the mappings are independently compared, once for the HPO to MP “translation” direction and once for the MP to HPO “translation” direction.

### Phenotype Similarity between Mouse Models and Diseases

**Figure 3:**
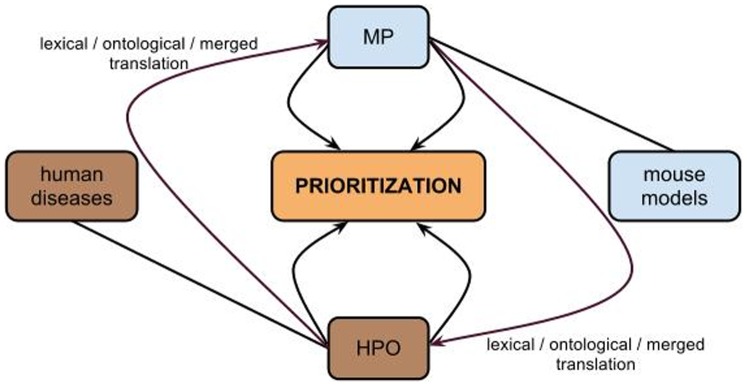
Highlights the applied transformation in our method. Our mappings are not symmetrical. Therefore, we can “translate” phenotype concepts in two directions: we can translate all mouse models into an HPO-based representation (using either the lexical, ontology-based or merged mapping approach), and we can translate all human diseases into an MP-based representation (using either of the mappings). When both mouse phenotypes and human diseases are represented using the same ontology, their similarity can be computed to suggest candidate disease genes. The original data obtained from OMIM (disease annotations in HPO) is illustrated with a brown color whilst the data obtained from MGI is illustrated with a light blue color. The purple arrows show the “translation” process using either the lexical, the ontological or the combined mapping. Once diseases and mouse models are represented using the same ontology, the prioritization based on a phenotype similarity will be calculated.

Based on the ontological mappings between the MP and HPO, we applied a measure of semantic similarity to compare experimentally derived phenotype descriptions of mice with the phenotypes that are associated with human diseases. Figure 3 provides an overview of the experimental setup of our approach. We used the phenotype annotations of mouse models available from the MGI database [22] and compared those to the phenotypes associated with diseases described in OMIM. To automatically compare the similarity between mouse and disease phenotypes, we converted either the mouse phenotypes into an HPO-based representation or the disease phenotypes into an MP-based representation. This transformation allowed us to perform a similarity-based comparison between phenotypes using either HPO or MP (also illustrated in Figure 3).

To identify the similarity 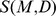 between a mouse model 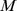 and a disease 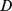, we used the Jaccard index between the phenotypes 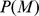 and 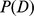 that are associated with 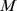 and 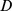:
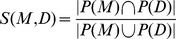


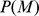
 and 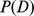 are sets of phenotypes that are either expressed using the MP or the HPO. Both sets are closed with respect to the taxonomy of either MP or HPO, i.e., if they contain a concept 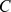 from MP or HPO they also contain all of 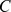
’s super-concepts. Due to the inclusion of the ontologies’ structure in the sets of phenotypes, 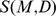 establishes a measure of semantic similarity [35], [36].

## Results

### Evaluation of Disease Gene Prioritizations

We computed the phenotype similarity between all mouse models in the MGI database and all disease phenotypes in OMIM. First, we utilized the three different mapping approaches (lexical, ontology-based, and a combination of both) between HPO and MP to “translate” human disease phenotypes into an MP-based representation, and compared their semantic similarity with mouse phenotypes based on MP. Second, we used the three mapping approaches to “translate” mouse phenotypes into an HPO-based representation and compared their semantic similarity with disease phenotypes based on HPO. As a result, we obtain six distributions of phenotype similarity values for each disease, three based on HPO’s structure, and another three for the similarity based on MP.

We individually applied the resulting similarities between mouse models and diseases to prioritize candidate genes for diseases. For this purpose, we assume that mouse models with a phenotype that is similar to a disease phenotype may be a model of that disease [13], [14], [21]. To evaluate this assumption, we compared our prioritization results against known gene–disease associations. To quantify how well our approach associates diseases with genes that may cause the disease, we generate and analyze the corresponding ROC curves. A ROC curve is a plot of the true positive rate as a function of the false positive rate. The Area Under Curve (AUC) is a quantitative measure of the performance of a classification task and is equivalent to the probability that a randomly chosen positive example is ranked higher than a randomly chosen negative one [37].

We performed the ROC analysis twice using either a set of known gene–disease associations in humans (OMIM’s MorbidMap) or using a set of gene–disease associations in mice (disease annotations available in the MGI database). In the absence of a large set of true negative gene–disease associations, we assume that only *known* gene–disease associations constitute *positive* examples while all *unknown* associations constitute *negative* examples.

**Figure 4:**
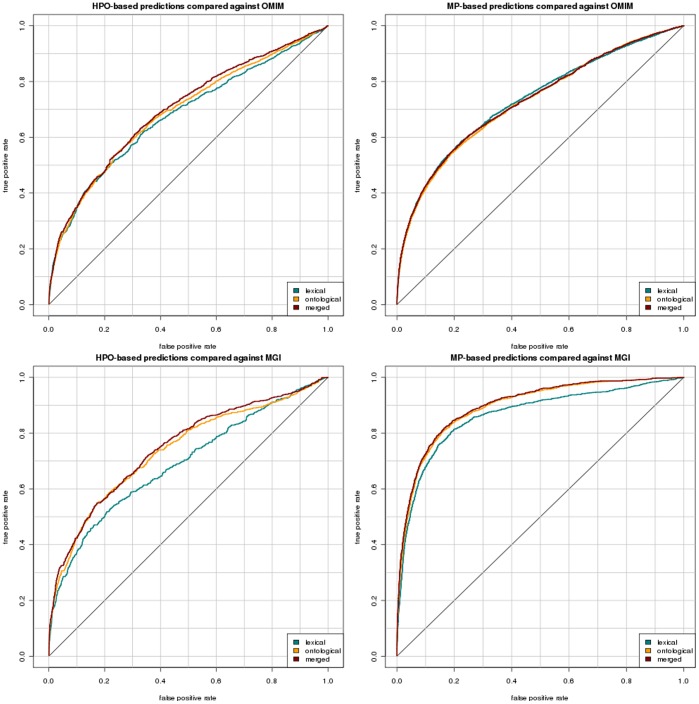
ROC curves resulting from our evaluation. The left panel includes all the results for “translating” mouse models from an MP representation to an HPO representation and performing the gene prediction in HPO. The right panel includes all the results for a “translation” of human diseases into an MP-based representation. Each plot shows the evaluation results using each of the three mappings: using lexical matching, using reasoning over ontologies, and the merged mappings. The two panels on the top are the results of the evaluation against OMIM and the two panels at the bottom are the results of the evaluation against MGI.

As a result, we obtained 12 ROC curves with their associated AUC values: we performed the similarity-based comparison based on HPO and based on MP for each of the three mapping approaches between MP and HPO and vice versa (based only on lexical matching, based only on reasoning over phenotype definitions, and based on the combination of both approaches), and evaluate the results against both MGI’s and MorbidMap’s gene–disease associations. Figure 4 illustrates the resulting ROC curves and table 2 shows the AUCs obtained for each.

**Table 2:** Areas Under Curve (AUC) measures for all gene prediction tasks.

based on mapping	HPO			MP		
	lexical	ontological	merged	lexical	ontological	merged
OMIM	0.678	0.690	0.700	0.732	0.727	0.730
MGI	0.691	0.737	0.748	0.864	0.895	0.899

The results in the first row show the AUC values for comparing against OMIM’s gene–disease associations, while the results in the second row are the AUC values when comparing against MGI’s gene–disease associations. Columns entitled *HPO* contain the results of the HPO-based gene prediction, whilst columns entitled *MP* contain the results of the MP-based gene prediction.

To determine the impact of the different mapping approaches on the task of gene prioritization, we determined the correlation between the prioritization results obtained using the lexical and the ontology-based mappings. Using Spearman’s rank correlation coefficient 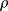, the correlation coefficients between the ranks of the positive examples using the lexical and ontology-based mapping approaches are 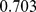 (HPO-based compared against OMIM’s gene–disease associations), and 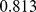 (MP-based compared against OMIM’s gene–disease associations), 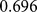 (HPO-based compared against MGI’s gene–disease associations) and 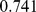 (MP-based compared against MGI’s gene–disease associations).

### Prioritizing Candidate Genes for Orphan Diseases

Based on the results of our quantitative evaluation, we can apply our method’s prioritization results to suggest candidate genes for orphan diseases. These can subsequently be studied in more detail or emphasized in large-scale mutagenesis projects such as the International Knockout Mouse Consortium [38]. To verify the potential of our method to correctly prioritize disease gene candidates, we have manually assessed the prioritization results obtained when calculating phenotype similarity based on MP and using the combination of lexical and ontology-based mappings (the scenario in which we achieved the highest AUC score).

**Figure 5:**
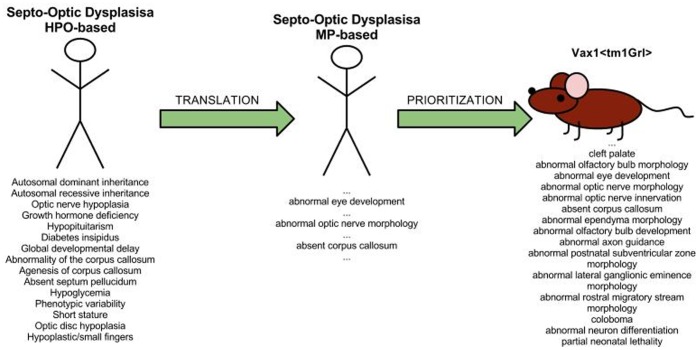
*Vax1* is one of the highest ranked mouse model for Septo-Optic Dysplasia. After combining both lexical and ontological mapping (illustrated in Figure 2), human diseases were “translated” with the combined mapping to an MP representation (results with highest AUC score). We manually verified some of the MP-based prioritization results (including Septo-Optic Dysplasia). The figure illustrates the original annotation for the disease based on HPO and its “translation” to MP. It also includes the annotations contained in MGI for mouse models with the *Vax1
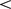
tm1Grl
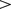
* (MGI:1859863) allele. To reduce the complexity of the figure, we did not include all annotations resulting from the “translation” of the disease annotations and after the enrichment of the mouse model annotations. A full list of all annotations is provided as supplementary material (supplementary file S1), also including other highly ranked mouse models for Septo-Optic Dysplasia.

For example, our method predicts knockouts of *Gdf6* (MGI:95689), *Marcks* (MGI:96907) and *Vax1* (MGI:1277163) on ranks 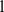, 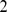 and 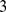 for *Septo-Optic Dysplasia* (SOD) (OMIM:#182230). Investigating further, we can suggest that *Vax1* could be a candidate gene for patients suffering from SOD. SOD is a disorder characterized by any combination of optic nerve hypoplasia, pituitary gland hypoplasia, and midline abnormalities of the brain, including absence of the corpus callosum and septum pellucidum [39]. *Vax1* mutations in mice share remarkable phenotypic similarities with SOD in humans as illustrated in Figure 5. For example, both the disease and the mouse models are annotated with *abnormal eye development* (MP:0001286), *abnormal optic nerve morphology* (MP:0001330), and *absent corpus callosum* (MP:0002196). Our results confirm a recent study in which *Vax1* has been suggested as a strong candidate gene for SOD when no *Hesx1* (MGI:96071) mutations are present [40]. Details on the steps involved in prioritizing *Vax1* for SOD, and parts of the input data we used (fully provided as supplemental material in supplementary file S1), are illustrated in Figure 5.

Furthermore, the genes our approach predicts on ranks 1 and 2 for SOD are *Gdf6* and *Marcks*. *Gdf6* has previously been identified to implicate ocular and skeletal abnormalities [41], in particular abnormalities of the coronal suture between bones in the skull [42], while deficiency of the *Marcks* protein in mice has been shown to result in an absence of the corpus callosum, cortical and retinal abnormalities [43]. Based on their phenotypic similarity to SOD (full information also provided as supplemental material in supplementary file S1), *Gdf6* and *Marcks* are promising novel candidates for genes involved in SOD.

The *HESX1* gene has been identified as a cause of SOD and hypopituitarism [44], [45], and we also identify a *Hesx1* model on rank 22 using our approach.

## Discussion

### Comparison to Related Work

The majority of the available systems for gene prioritization follow a “guilt-by-association” approach [15] and use information about known genes–disease associations to identify genes that are similar (with respect to a wide variety of features) to known causal genes for a disease. The features that are used for determining similarity in these tasks include GO annotations, phenotypes, information about gene expression, gene regulation, sequence, homology, interactions and pathway data as well as literature information [15]. Methods following a “guilt-by-association” approach require prior knowledge about the molecular origins of a genetic disorder and can not be applied when such information is not available. An approach based exclusively on comparisons of phenotypes requires no prior knowledge about molecular mechanisms underlying a disease and can therefore be applied to diseases for which the phenotype is known, regardless of whether genetic causes for the disease are already known.

After pioneering studies have shown that comparisons of phenotypes can reliably prioritize candidate disease genes [9], [13], two recent approaches, PhenomeNET [14] and MouseFinder [46], applied phenotype-based gene prioritization in large scale to data from mouse model experiments.

PhenomeNET implements the first large-scale application of gene prioritization based on cross-species phenotype similarity applied to phenotypes of yeast, fly, worm, fish, mouse and human diseases. Using the whole dataset consisting of phenotypes in six species, PhenomeNET achieves an AUC in a ROC analysis for prioritizing gene–disease associations of 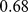 (compared against a combination of MorbidMap’s and MGI’s gene–disease associations as positive instances). To compare our results to the PhenomeNET approach, we restricted the PhenomeNET dataset to the mouse models and the diseases we used in our approach and separately evaluated the prioritization results against both, OMIM’s and MGI’s gene–disease associations. When comparing against OMIM’s associations, the AUC of PhenomeNET is 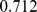, and when comparing against MGI’s associations the AUC is 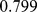.

Our approach (MP-based, using a combination of both lexical and ontology-based mappings) achieves a significantly improved performance over PhenomeNET (comparing against OMIM’s associations: 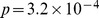, comparing against MGI’s associations: 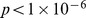, one-tailed Student’s *t*-test). The main difference of our approach to PhenomeNET is the similarity computation, which we performed using only a single phenotype ontology (either MP or HPO), while PhenomeNET uses a combination of five different phenotype ontologies for the computation of the semantic similarity. The inclusion of multiple, often redundant (i.e., equivalent) phenotypes classes introduces additional noise that affects the resulting similarity values. Furthermore, we utilize lexical mappings in addition to the ontology-based mappings, while PhenomeNET relied on ontology-based mappings exclusively. PhenomeNET also uses a weighted Jaccard index as a similarity measure while we do not employ weights. We intend to evaluate the impact of differences in semantic similarity measures as future research.

Another implementation of using mouse models to prioritize gene candidates for human genetic disorders is the *MouseFinder* [46]. Similar to PhenomeNET, MouseFinder relies on mappings generated via definitions in ontologies and bases the computation of semantic similarity on a combination of the HPO and MP. In MouseFinder, several similarity measures are implemented and the results using either measure are compared, finding that a similarity measure based on a weighted Jaccard index achieves the highest recall in the task of gene prioritization. In total, within the first 500 ranks, MouseFinder reports a recall of 58% when compared against OMIM’s gene–disease associations and 65% when compared against MGI’s associations. Since ranks are shared by multiple mouse models in MouseFinder (i.e., multiple mouse models with the same similarity values share a rank), we cannot derive the precision of the MouseFinder approach and therefore lack the means for a direct comparison.

### Cross-species Comparison

The main difference of our method to previous approaches for phenotype-based gene prioritization is the inclusion of lexical matches and the use of single, species-specific phenotype ontologies to compute phenotypic similarity. We observe significant differences in the performance of our method depending on which mapping method between MP and HPO we apply, which ontology we use to compute semantic similarity, and against which set of gene–disease associations we perform our evaluation.

Our first observation is that the performance of our approach is usually better when using ontology-based mappings than when using lexical mappings alone. In one case, using MP-based phenotypic similarity evaluated against OMIM, lexical matching (AUC 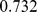) performs slightly better than ontology-based matching (AUC 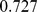). It seems surprising that the lexical matching of 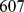 concepts can almost match ontology-based mappings (based on more than 10,000 formal concept definitions in both the MP and HPO) when applied to the task of gene prioritization. A possible explanation lies in the annotation depth of mouse models in the MGI database as well as the depth of concepts that match exactly between the MP and HPO. On average, mouse models in the MGI are annotated at a depth of 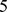 in the MP [47]. The concepts that lexically match exactly between the HPO and MP, however, are mostly specialized, clinical terms that are used for annotating disease-related phenotypes in OMIM. These terms denote complex concepts that carry substantial information about a disorder. As a result of their complexity, they are often not formally defined and would therefore not map completely across species when using ontology-based mappings. If an appropriate MP concept can be identified, all mouse models that are annotated with it or any of its super-classes will share features with the clinical term and therefore have some similarity to the disease that includes the complex clinical phenotype. On the other hand, mouse models are rarely annotated with these clinical terms, and mappings through lexical matching may not identify a single matching class from HPO. While mappings through lexical matching may prefer one direction (from HPO to MP) due to the differences in annotation between OMIM and MGI’s mouse models, we observe no such bias for mappings generated through automated reasoning over phenotype class definitions.

However, computing similarity within MP performs always better than computing similarity within the HPO. This may be an indication that either the structure or the content of the MP is more suitable for our particular application (i.e., the prioritization of mouse models) than the structure and content of the HPO. At the minimum, our method provides an objective, quantitative measure of the performance of both ontologies and their definitions with regard to phenotype-based gene prioritization of mouse models, and this measure may be used to further develop and improve the ontologies.

Finally, we observe a significant difference in the performance of our method depending on whether we evaluate against MGI’s or OMIM’s gene–disease associations. When evaluating our predictions against OMIM’s gene–disease associations, we achieve, at best, an AUC of 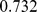 (using lexical mapping, MP-based similarity computation), while we obtain up to 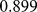 when evaluating against MGI’s gene–disease associations (merged mapping, MP-based similarity computation). Furthermore, mappings through lexical matching perform similar to ontology-based mapping when evaluating against OMIM, while we observe a notable decline in performance when evaluating against MGI’s disease annotations. The magnitude of the difference between both data sets may be indicative of different guidelines in the amount of evidence that is required to assert a gene–disease relation in both databases.

### Future Directions for Phenotype Analysis

Our approach is currently limited by the quantity and quality of cross-species mappings between phenotype ontologies. Possible further extensions of our approach could be the application of less restrictive lexical matching algorithms or additional approaches to ontology mapping [48] to increase the number of matched concepts. In particular, we currently use exact matching between phenotype terms to derive lexical mappings between the HPO and MP. A possible future extension is to incorporate less conservative matches such as those derived from stemming algorithms. Furthermore, the mappings could also be improved by investigating better algorithms to integrate both the lexical and ontological mapping, allowing, for example, for partial matches that map to subclass assertions instead of statements of equivalence.

Another future extension is to apply our method to other resources such as OrphaNet [49] or DECIPHER [50] as well as other model organism databases. As a first step in this direction, we have incorporated our results into the PhenomeNET method, where the results are available via the PhenomeBrowser [14].

## Supporting Information

File S1. SOD-supplement.ods The three highest ranked mouse genes for Septo-Optic Dysplasia are Gdf6, Marcks, and Vax1. Supporting file S1 contains the original MP annotations of the three highest ranked mouse alleles corresponding to the beforementioned genes and also provides the original HPO annotations for the disease. Furthermore, it also contains the MP annotations for Septo-Optic Dysplasia after applying the combined lexical and ontological mapping.
(ODS)
